# Potentiating effect of AMD3100 on bone morphogenetic protein-2 induced bone regeneration

**DOI:** 10.1186/s40902-024-00431-y

**Published:** 2024-06-17

**Authors:** Gyu-Jo Shim, Chung O. Lee, Jung-Tae Lee, Hong-Moon Jung, Tae-Geon Kwon

**Affiliations:** 1 Department of Oral & Maxillofacial Surgery, School of Dentistry, Kyungpook National University, and Institute for Translational Research in Dentistry, Kyungpook National University, Daegu, Republic of Korea; 2 Department of Radiologic Technology, Daegu Health College, Daegu, Republic of Korea; 3 Department of Oral & Maxillofacial Surgery, School of Dentistry, Kyungpook National University, and Kyungpook National University Institute for Translational Research in Dentistry, 2177 Dalgubeol-daero, Jung-Gu, Daegu, 41940 Republic of Korea

**Keywords:** AMD3100, Bone morphogenetic protein 2, Bone regeneration

## Abstract

**Background:**

AMD3100, a CXCR4 antagonist, is currently prescribed for activating the mobilization of hematopoietic stem cells. Recently, AMD3100 was shown to potentiate bone morphogenetic protein-2 (BMP-2)-induced bone formation by stimulating the trafficking of mesenchymal cells. However, optimization of the strategic combination of AMD3100 and BMP-2 has not yet been clearly established. The purpose of this study was to evaluate the effect of AMD3100 on BMP-2-induced bone regeneration in vitro and in a mouse calvarial defect healing model.

**Methods:**

In vitro osteoblastic differentiation and cell migration after sequential treatments with AMD3100 and BMP-2 were analyzed by alkaline phosphatase (ALP) activity, ALP staining, and calcium accumulation. Migration capacity was evaluated after treating mesenchymal cells with AMD3100 and/or BMP-2. A critical-size calvarial defect model was used to evaluate bone formation after sequential or continuous treatment with AMD3100 and BMP-2. The degree of bone formation in the defect was analyzed using micro-computed tomography (micro-CT) and histological staining.

**Results:**

Compared with single treatment using either AMD3100 or BMP-2 alone, sequential treatment with AMD3100 followed by BMP-2 on mesenchymal cells increased osteogenic differentiation. Application of AMD3100 and subsequent BMP-2 significantly activated cell migration on mesenchymal cell than BMP-2 alone or AMD3100 alone.

Micro-CT and histomorphometric analysis showed that continuous intraperitoneal (IP) injection of AMD3100 resulted significantly increased new bone formation in BMP-2 loaded scaffold in calvarial defect than control groups without AMD3100 IP injection. Additionally, both single IP injection of AMD3100 and subsequent BMP-2 injection to the scaffold in calvarial defect showed pronounced new bone formation compared to continuous BMP-2 treatment without AMD3100 treatment.

**Conclusion:**

Our data suggest that single or continuous injection of AMD3100 can potentiate BMP-2-induced osteoblastic differentiation and bone regeneration. This strategic combination of AMD3100 and BMP-2 may be a promising therapy for bone regeneration.

## Background

Bone tissue exhibits the dynamic processes of formation and resorption throughout life and is one of the few organs that can be regenerated in adulthood. However, the outcomes of reconstruction used to bone defects are not always predictable even with autogenous bone graftings, which is regarded as the gold standard among bone regeneration procedures [1]. Autogenous bone contains only small amounts of pluripotent stem cells and displays slow proliferation rates and limited potential for new bone formation [2]. Therefore, the application of various cytokines, such as transforming growth factors [3], bone morphogenetic proteins (BMPs) [4], fibroblast growth factors [5], and insulin-like growth factors [6] in bone regeneration have studied. These growth factors stimulate stem cells or progenitor cells to proliferate or differentiate similar to the surrounding cells. Among them, BMPs have been reported to induce new bone formation at both ectopic and orthotopic sites [4, 7–9]. BMP-2 is a strong inducer of new bone formation and plays a role in the homing of osteoblast progenitor cells [10]. It also induces angiogenesis and promotes the osteogenic differentiation of mesenchymal stem cells [8, 11]. Therefore, BMP-2 is approved by FDA and currently widely used for clinical settings for bone regeneration in maxillofacial and orthopedic fields. But the side effects such as swelling, seroma or potential risk of cancer were suggested, which was related with high dosage application of BMP-2 [12]. It had been reported that relatively high concentrations of BMP-2 are required to ensure effectiveness in clinical settings [13], which may induce undesired side effects, such as apoptosis, increased vascularity, fibroblast infiltration, inflammatory reactions, and other catabolic effects. Moreover, the increase of concentration does not incudes dose-dependent bone regeneration [14]. Therefore, the utilization of other chemokines or cytokines has been proposed to overcome these problems. At the same time, combination of various growth factors or mesenchymal cells was suggested to have synergistic effect for bone regeneration [15–21].

Recently, the stromal cell-derived factor-1 (SDF-1)/ circulating cysteine-C motif chemokine receptor 4 (CXCR4) axis has been reported to has a role in the osteogenic process [16–18, 22]. SDF-1/CXCR4 signaling is critical in maintaining tissue homeostasis. Several previous studies have suggested that SDF-1 signaling is essential in marrow stromal cell (MSC)-mediated tissue repair and regeneration of various organs including bone [19–21, 23–27]. SDF-1 is derived from the injured periosteum and promotes the migration of MSCs to the damaged sites, subsequently inducing cartilaginous bone formation after binding with circulating CXCR4-expressing progenitor cells [15, 28]. Paradoxically, recent studies have shown that the CXCR4 antagonist AMD3100 also promotes tissue regeneration by regulating the homing of MSCs [29–32].

AMD3100 (1, 1—[1, 4'—phenylenebis—(methylene)]—bis—1, 4, 8, 11—tetraazacyclotetradecane) is a small bicyclam molecule that reversibly and selectively blocks binding of SDF-1 to its receptor CXCR4. AMD3100 was originally developed as an anti-HIV agent shown to be effective in inhibiting the replication of HIV in vitro at nanomolar concentrations [33, 34]. However, the results of initial clinical trials involving the use of AMD3100 for AIDS treatment revealed the unexpected side effects due to increased white blood cell counts [33]. AMD3100 was subsequently developed and marketed as a stem cell mobilizer (plerixafor), and additional studies demonstrated that it is well tolerated by healthy volunteers and produces minimal and reversible side effects, such as injection site pain, erythema, headache, nausea, and abdominal distension or cramping [35, 36]. In December 2008, AMD3100 was approved by the FDA for hematopoietic stem and progenitor cell (HSPC) mobilization in combination with granulocyte colony-stimulating factor (G-CSF) in patients with non-Hodgkin's lymphoma (NHL) or multiple myeloma undergoing autologous bone marrow transplantation. Since then, AMD3100 has been used in patients with NHL or multiple myeloma who failed to mobilize HSPCs with both G-CSF alone or G-CSF plus chemotherapy [37].

Previous studies have revealed that AMD3100 mobilizes endothelial progenitor cells and increases the number of circulating angiogenic cells [29, 30]. These mobilized cells displayed greater proliferative capacity and angiogenic potential than do steady-state circulating cells. In preclinical studies, these cells were able to home to the sites of vascular injury, stimulate angiogenesis, and improve revascularization. Therefore, the use of AMD3100 might be a beneficial therapeutic strategy to stimulate revascularization after tissue injury [37]. Recently, AMD3100 has been shown to potentiate BMP-2 induced bone formation by stimulating the trafficking of mesenchymal cells to ectopic implant sites in the rats [18]. However, the combined use of AMD3100 and BMP-2 has not been optimized, and there are only a few studies that have investigated the biological mechanism behind the combination of AMD3100 and BMP-2 applied to in vivo bone defect regeneration [16, 22, 28].

We hypothesized that the administration of AMD3100 and BMP-2 may increase osteogenesis and bone regeneration in animal model, and concomitant or sequential application can be beneficial to bone formation. The purpose of this study was to evaluate the potentiating effect of AMD3100 on BMP-2-induced intramembranous bone regeneration in vitro and in a mouse calvarial defect-healing model.

## Methods

### Cell culture

The biological effects of AMD3100 or BMP-2 on mesenchymal cells were evaluated with pluripotent mesenchymal progenitor C3H10T1/2 cells and myoblastic C2C12 cells which were acquired from the American Type Culture Collection (ATCC, Manassas, VA, USA). Mouse bone marrow stromal cells (BMSCs) were harvested according to the previous report [38] and the passage 2–3 were used in experiments. C3H10T1/2, C2C12 cells, and BMSC were cultured at 37 °C in a humidified atmosphere of 5% CO_2_ in Dulbecco's modified Eagle's medium (DMEM; Lonza Group Ltd., Basel, Switzerland) supplemented with 10% fetal bovine serum (FBS; Life Technologies, Carlsbad, CA), 100 U/mL penicillin, 100 mg/mL streptomycin sulfate and 2 mM glutamine. Recombinant BMP-2 proteins were purchased from PeproTech Inc. (New Jersey, USA) and Daewoong Pharmaceutical company (Seoul, Korea), respectively. L-ascorbic acid, β-glycerophosphate and AMD3100 were all purchased from Sigma-Aldrich (#A5602, St. Louis, MO, USA).

### Alkaline phosphatase (ALP) activity assay

To evaluate the effect of AMD3100 and BMP-2 on Alkaline phosphatase (ALP) activity, C3H10T1/2 cells were plated in 12-well plates (Corning® Costar®; Sigma-Aldrich) at a concentration of 5 × 10^5^ cells/well (1.3 × 10^5^ cells/cm^2^) and cultured in osteogenic media (20% FBS with 1% antibiotics in DMEM, 10 mM β-glycerophosphate and 50 µg/ml l-ascorbic acid 2-phosphate) for 9 days. One day after seeding, the media were replaced every 2 days with the addition of AMD3100 (100 µM) and/or BMP-2 (0.5 µg/mL). For example, A-B-B-B indicated that the cells were cultured in media containing AMD3100 for the first 2 days and then replaced with BMP-2-containing media on the 3^rd^, 5^th^, and 7^th^ days, followed by final cell harvesting on the 9^th^ day after cell seeding. AB-AB-AB-AB group represents concomitant application of both AMD3100 and BMP2 when the media was replaced. The cells were then harvested and lysed using ProteoJET lysis buffer (Fermentas, St. Leon-Rot, Germany). The cell extracts were incubated with p-nitrophenyl phosphate substrate (Sigma-Aldrich) in a 0.05 M glycine buffer containing 0.05 mM MgCl_2_ (pH 10.5) at 37 °C for 30 min. ALP activity was evaluated by measuring absorbance at 405 nm using a microplate reader (Infinite® M200, Tecan U.S., Inc. Mannedorf, Switzerland) and normalized to the total protein content of cell lysates.

### ALP staining assay

ALP staining was performed using an ALP detection kit (Sigma-Aldrich), according to the manufacturer’s instructions. Twelve-well plates with osteogenic media comprising 100 uM AMD3100 and/or 0.5 µg/mL BMP-2, both of which were replaced every 2 days for a total of 8 days (similar to the procedure for the ALP activity assay), were seeded with C3H10T1/2 cells at a concentration of 5 × 10^4^ cells/well (5.3 × 10^4^ cells/cm^2^) in triplicate. Subsequently, each well containing the cells was washed with phosphate-buffered saline (PBS). The fixation solution was applied for 45 s. After reaction with ALP substrate, the solution was removed and the cells were washed three times using PBS. The ALP-positive cells were identified by red staining.

### Calcium accumulation assay

C3H10T1/2 cells were seeded on 12 well plates (5 × 10^4^ cells/well) and were incubated in 5% FBS DMEM osteogenic media with 100 µM AMD3100 and/or 0.5 µg/ml BMP-2 for 8 days. Calcium levels were determined colorimetrically using a calcium assay kit (Calcium Colorimetric Assay Kit, Abcam, and Cambridge, MA, USA) by following the manufactures’ protocol. The optical density was measured at 595 nm using a microplate reader (Infinite® M200, Tecan U.S., Inc. Mannedorf, Switzerland) and normalized to total protein content.

### Cell migration assay

The effects of AMD3100 and/or BMP-2 treatment on cell migration were evaluated with a transwell migration assay as previously described [38]. C2C12 cells and BMSC (3 × 10^5^ cells/well) were suspended in 100 µL serum-free DMEM and loaded into upper wells (Corning® Transwell™; Sigma-Aldrich). After the serum-free media containing 1 µM AMD3100 or 0.1 µg/mL BMP-2 were added in the upper chamber for 24 h with serum free media, AMD3100 and/or BMP-2 were added the lower chamber containing 2% FBS-containing media to for a further 24 h. For migration assay, cells were washed with 600 µL PBS and fixed with 600 µL ethanol for 10 min. After washing again with 600 µL PBS, the cells remaining on the inner surface of the upper chambers were removed with cotton swabs. For example, to demonstrate the effect of the sequential treatment of AMD3100 and subsequent BMP-2, the medium in the upper chamber was replaced with 1 µM of AMD3100 in 300 mL of serum-free DMEM and incubated for 1 h, following which the medium in the lower chamber was replaced with 0.1 ug/mL BMP-2 (AMD3100 → BMP-2, marked as A → B in the figure).

The chambers were stained with 600 µL 0.2% crystal violet (Junsei Chemical Co. Ltd, Tokyo, Japan) for 30 min at room temperature, and the cells in four random visual fields were counted (100 × magnification).

### Colony-forming unit (CFU) assay after AMD3100 injection

To analyze the in vivo effect of AMD3100 on hematopoietic progenitor cell mobilization, AMD3100 or PBS was administered by intraperitoneal (IP) injection at a concentration of 2 mg/kg, which was determined based on a previous study that reported the effect of optimal mobilization of HSPCs 1 h after AMD3100 injection [39]. For the CFU assay, peripheral blood (PB) was obtained 1 h after the injection using the cardiac puncture method. Single-cell suspensions of PB were obtained after ammonium chloride lysis to exclude the red blood cells. The centrifuged cells were counted using with 3% acetic acid with methylene blue and were plated into 35-mm dishes (1 × 10^5^ cells / dish). The cells were incubated in MethoCult media (GF M3434, StemCell Technologies, Vancouver, Canada), and the hematopoietic colonies were counted and scored after incubation for 14 days. The number of hematopoietic CFU cells were analyzed.

### Mouse critical-size calvarial defect model: effect of AMD3100 and BMP-2

Seven-week-old female C57BL/6 mice (Hyochang Science, Daegu, Korea) were used as the critical-size calvarial defect models to evaluate the efficacy of combined treatment of AMD3100 and BMP-2 treatment for orthotopic bone formation. All the animal experiments were approved by the Institutional Animal Care and Use committee Kyungpook National University (authorization number; KNU 2010–30). PBS or AMD3100 (2 mg/kg; Sigma-Aldrich) was administered by IP injection before surgical implantation. One hour later, all the animals (*n* = 32) were anesthetized by an IP injection of ketamine (100 mg/kg) and xylazine (5 mg/kg). Subsequently, the scalp was opened at the midline exposing the underlying calvaria. The calvarial defect was made on the parietal bone of mice using a 6 mm-diameter dental trephine bur under PBS irrigation. The dura mater was preserved without damage. After removing the 6 mm-diameter calvarial bone segment, a 6 mm collagen scaffold (Teruplug, Terudermis Olympus Terumo Biomaterials Co., Japan) was prepared. The PBS or BMP-2 (5 µg in 50uL of PBS) loaded scaffold was implanted in the defect area. The overlying soft-tissue wound was sutured immediately with 4–0 silk.

To evaluate the effect of continuous injection of AMD3100 and sequential treatment of AMD3100 and BMP-2 in bone regeneration, the two experimental settings were used in mouse calvarial defect model. Initially, BMP-2- or PBS-loaded collagen scaffolds were implanted into the calvarial defects. Overall experimental settings were shown in Table 1.

**Table 1:** Experimental settings for AMD3100 and/or BMP-2 administration

**Experiment 1**	**Continuous injection of AMD3100**				
Groups	Injection	At the time of operation	Day 3 after operation	Day 6 after operation	Day 28 after operation
C–C-C	IP injection Cranial defect	PBS injection PBS-loaded scaffold	PBS injection -	PBS injection -	Sacrifice
A-A-A	IP injection Cranial defect	AMD3100 PBS-loaded scaffold	AMD3100 -	AMD3100 -	Sacrifice
B-C–C	IP injection Cranial defect	PBS injection BMP-2-loaded scaffold	PBS injection -	PBS injection -	Sacrifice
AB-A-A	IP injection Cranial defect	AMD3100 BMP-2-loaded scaffold	AMD3100 -	AMD3100 -	Sacrifice
**Experiment 2**	**Sequential treatment of AMD3100 and BMP-2**				
Groups	Injection	At the time of operation	Day 3 after operation	Day 6 after operation	Day 28 after operation
C–C-C	IP injection Cranial defect	PBS injection PBS-loaded scaffold	PBS injection -	PBS injection -	Sacrifice
A-C–C	IP injection Cranial defect	AMD3100 injection PBS-loaded scaffold	PBS injection -	PBS injection -	Sacrifice
C-B-B	IP injection Cranial defect	PBS injection PBS-loaded scaffold	PBS injection BMP-2 injection	PBS injection BMP-2 injection	Sacrifice
A-B-B	IP injection Cranial defect	AMD3100 PBS-loaded scaffold	PBS injection BMP-2 injection	PBS injection BMP-2 injection	Sacrifice

To evaluate the effect of continuous injection of AMD3100 and sequential treatment of AMD3100 and BMP-2 in bone regeneration, the two experimental settings were used in mouse calvarial defect model. Initially, BMP-2- or PBS-loaded collagen scaffolds were implanted into the calvarial defects. In experiment 1, IP injection of AMD3100 or PBS were performed on the indicated time points. In experiment 2, PBS or AMD3100 was injected IP one hour before the calvarial defect fabrication. PBS-loaded scaffold were implanted to the cranial defect. Then BMP-2 or PBS were injected to scaffold-implanted calvarial defect on day 3 and 6

*C* Control, *A* AMD3100 (2 mg/kg; Sigma-Aldrich), *B* BMP-2 (Bone morphogenetic progtein-2, 5 µg in 50uL of PBS), *PBS* Phosphate buffered saline, *IP* Intraperitoneal

The first animal experiment was intended to evaluate the effect of continuous injection of AMD3100 on BMP-2-induced bone formation, for which PBS or AMD3100 (2 mg/kg) was administered by IP injection on postoperative days 3 and 6. The animals were divided into the following four groups (*n* = 4 / group); group C–C-C, the PBS-loaded collagen scaffold was inserted in the calvarial defect and PBS was administrated by IP injection on postoperative days 3 and 6 after the surgery; group A-A-A, the PBS-loaded scaffold was implanted, and AMD3100 was administered by IP injections 1 h before the surgery with additional injections on days 3 and 6; group B-C–C, the BMP-2-loaded scaffold was inserted, and PBS was administered by IP injections on days 3 and 6; group AB-A-A, the BMP-2-loaded scaffold was implanted, and AMD3100 was administered by IP injections 1 h before the surgery with additional injections on postoperative days 3 and 6.

The second animal experiment was performed to evaluate the effect of sequential treatment, for which BMP-2 was locally administered in the calvarial defect after AMD3100 IP injection.

The animal was divided into four groups (*n* = 4 / group); group C–C-C, the PBS-loaded scaffold was implanted in the calvarial defect, and PBS was administered by IP injections on postoperative days 3 and 6; group A-C–C, the PBS-loaded scaffold was implanted in the calvarial defect, AMD3100 was administered by IP injections 1 h before the surgery, and PBS was administered by IP injections on days 3 and 6; group C-B-B, the PBS-loaded scaffold was implanted in the calvarial defect, and BMP-2 was injected into the scaffold on days 3 and 6; group A-B-B, AMD3100 was administered by IP injections 1 h before implantation of the PBS-loaded scaffold in the calvarial defect, and BMP-2 was injected locally into the scaffold on postoperative days 3 and 6.

After 28 days, the mice were euthanized by CO_2_ inhalation and the calvarial specimens were harvested for histology and micro-computed tomography (micro-CT) analyses.

### Micro-CT analysis of bone formation

New bone formation at the calvarial bone defect site were evaluated with micro-CT. The mouse calvarial specimens containing the PBS or BMP-2-loaded scaffold were excised, trimmed and fixed in 4% paraformaldehyde for 24 h at room temperature. They were then transferred into PBS and stored at 4 °C until the time for micro-CT analysis. The specimens were scanned using micro-CT (Skyscan 1072–32, Bruker Corporation, Kontich, Belgium) under an aluminum filter measuring 0.5 mm using an X-ray voltage of 50 kV, anode current of 200 mA, isotropic voxel size of 10 μm, and exposure time of 1.2 s with a pixel size of 2.5 μm. Serial axial images were reformatted to generate three-dimensional image reconstructions using TomoNT software (Skyscan software). The regenerated bone volume (mm^3^), trabecular number (1/mm), trabecular separation (mm) and trabecular thickness (mm), were measured in each group.

### Histological evaluation of bone formation

After taking the micro-CT measurements, the specimens were fixed in cold 4% paraformaldehyde solution for 10 days and then decalcified in 18% ethylenediaminetetraacetic acid (EDTA). The samples were then dehydrated and embedded in paraffin. The sections (10 μm in thickness) were subjected to hematoxylin and eosin (H & E) and trichrome staining. Areas of newly formed bone (measured in mm^2^) were analyzed histomorphometrically using i-solution software (Image & Microscope Technology, Korea).

### Statistical analysis

All the experiments were repeated two to three times independently, with four samples included in each treatment group. All data were expressed as mean ± standard deviation (SD). The differences between different test conditions were compared by the Tukey–Kramer post-hoc test, with the level of significance was set at *p* < 0.05. All the experiments were repeated at least in triplicate.

## Results

### Effect of AMD3100 on HSPC mobilization to blood

**Fig. 1:**
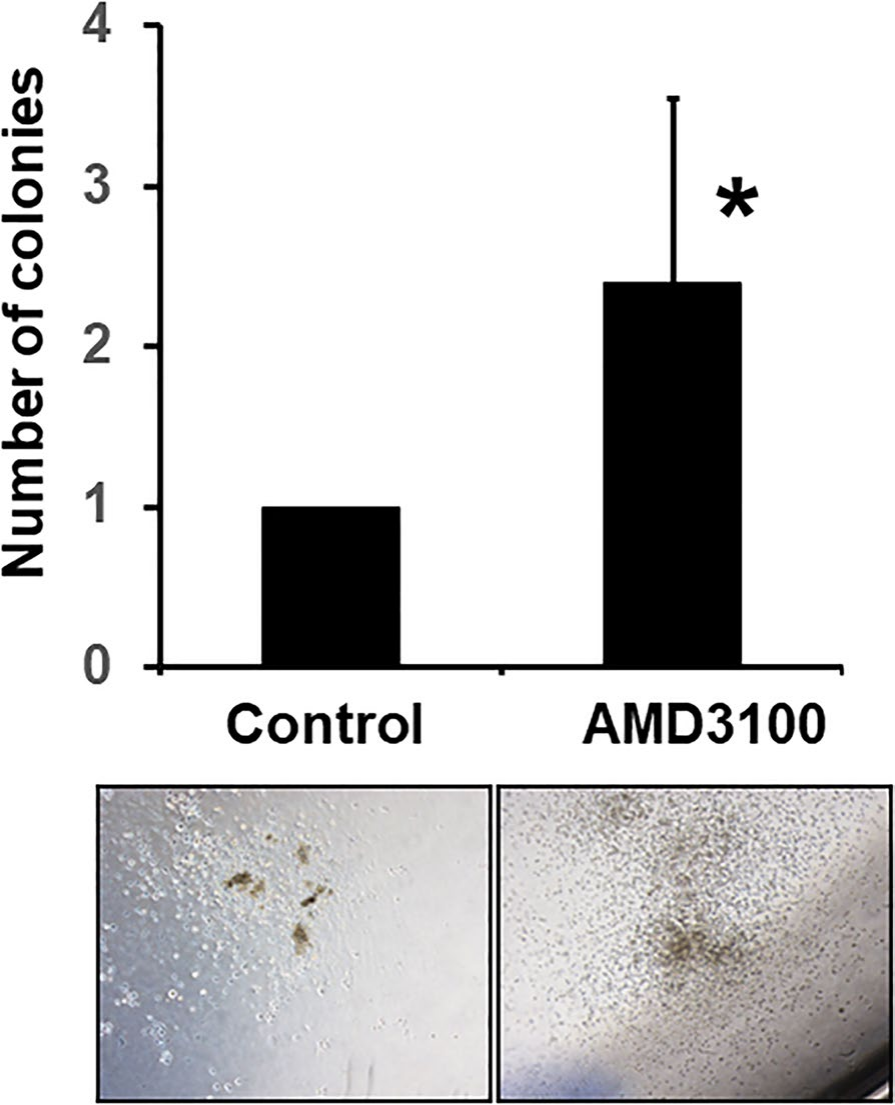
Colony-forming unit (CFU) assay after AMD3100 injection. AMD3100 was administered by intraperitoneal (IP) injection (2 mg/kg concentration), and peripheral blood (PB) was extracted. The cells were incubated in a semi-solid medium and hematopoietic colonies were counted and scored after incubation for 14 days. Compared with the non-AMD3100-treated controls, the AMD3100-treated samples exhibited significantly enhanced mobilization of hematopoietic stem and progenitor cells (HSPCs) to the blood. Data represent mean ± SD (*p < 0.05)

To determine the effect of AMD3100 on PB, CFU assay was performed after AMD3100 injection. Colonies comprising at least 30 cells were counted. The results showed that compared with the non-AMD3100-treated controls, the AMD3100-treated samples exhibited significantly enhanced mobilization of HSPCs to the blood (*p < 0.05) (Fig. 1).

### In vitro effect of AMD3100 in BMP-2 induced osteogenic differentiation

**Fig. 2:**
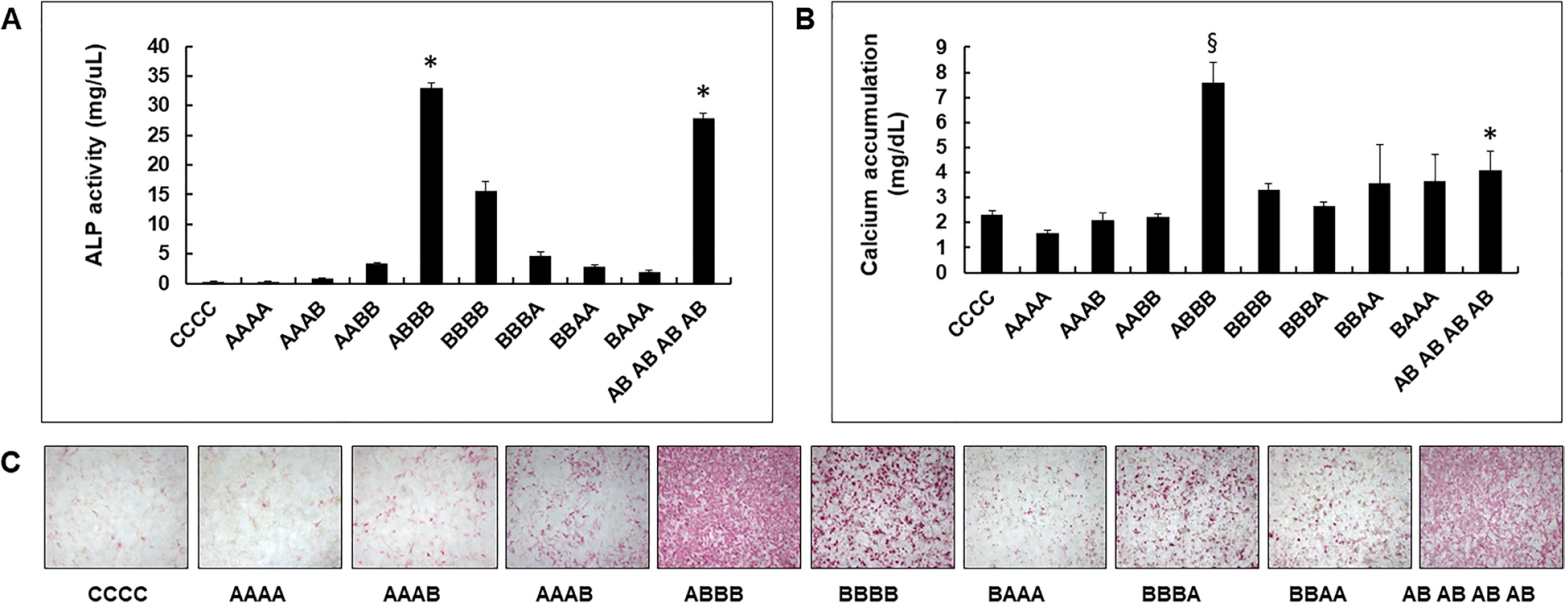
In vitro effect of AMD 3100 on BMP-2- induced osteoblastic differentiation. C3H10T1/2 cells (1 × 10^4^ cells/well) were incubated in osteogenic media and replaced every 2 days with or without AMD3100 (**A**, 100 μM) and/or BMP-2 (**B**, 0.5 µg/mL). The control was marked as **C** when the cells were grown only under the osteogenic media. For example, in the A-B-B-B group, the cells were cultured in media containing AMD3100 for the first 2 days and then replaced with BMP-2-containing media on the 3^rd^, 5^th^, and 7^th^ days, followed by final cell harvesting on the 9^th^ day after cell seeding. In the AB-AB-AB-AB group, concomitant application of both AMD3100 and BMP-2 was performed when the media was replaced. After treatment with the indicated supplements for 8 days, osteoblastic differentiation was analyzed by measuring alkaline phosphatase (ALP) activity (Upper left), calcium accumulation (Upper right), and ALP staining (Lower). The ALP-positive cells were stained red. Data indicate mean ± SD. (Upper left) *Significantly different from the other groups except A-B-B-B and A-B-AB-AB-AB group (all *p* < 0.05). (Upper right) §Significantly different from the other groups (all *p* < 0.05); and *significant difference between C–C-C–C, A-A-A-A, A-A-A-B, A-A-B-B, and A-B-B-B groups (all *p* < 0.05)

We used an ALP in vitro activity assay to determine the ability of sequentially applied AMD3100 and BMP-2 to induce osteogenic differentiation in C3H10T1/2 cells. Initial AMD3100 treatment and continuous BMP-2 application afterward (group A-B-B-B) was found to be the most effective regimen for osteogenic differentiation and calcium accumulation. However, prolonged application of AMD3100 seemed to inhibit BMP-2-induced osteogenesis (groups A-A-A-B and A-A-B-B). Initial BMP-2 treatment and subsequent AMD3100 treatment did not enhance more osteogenic differentiation (groups B-B-B-A, B-B-A-A, and B-A-A-A) (*p* < 0.05). Moreover, long-term AMD3100 treatment after BMP-2 further inhibited osteoblastic differentiation. Concomitant, continuous BMP-2 and AMD3100 treatment (group AB-AB-AB-AB) also increased osteogenic differentiation, which was comparable to initial, single AMD300 application followed by BMP-2 application (group A-B-B-B) (*p* < 0.05), as demonstrated by ALP activity and staining. However, calcium accumulation in the AB-AB-AB-AB group was not dramatically high when compared with that in the A-B-B-B group. There were significant differences between C–C-C–C, A-A-A-A, A-A-A-B, A-A-B-B, and A-B-B-B groups in calcium accumulation (all *p* < 0.05) (Fig. 2).

### In vitro cell migration assay in combined treatment of AMD3100 and BMP-2

**Fig. 3:**
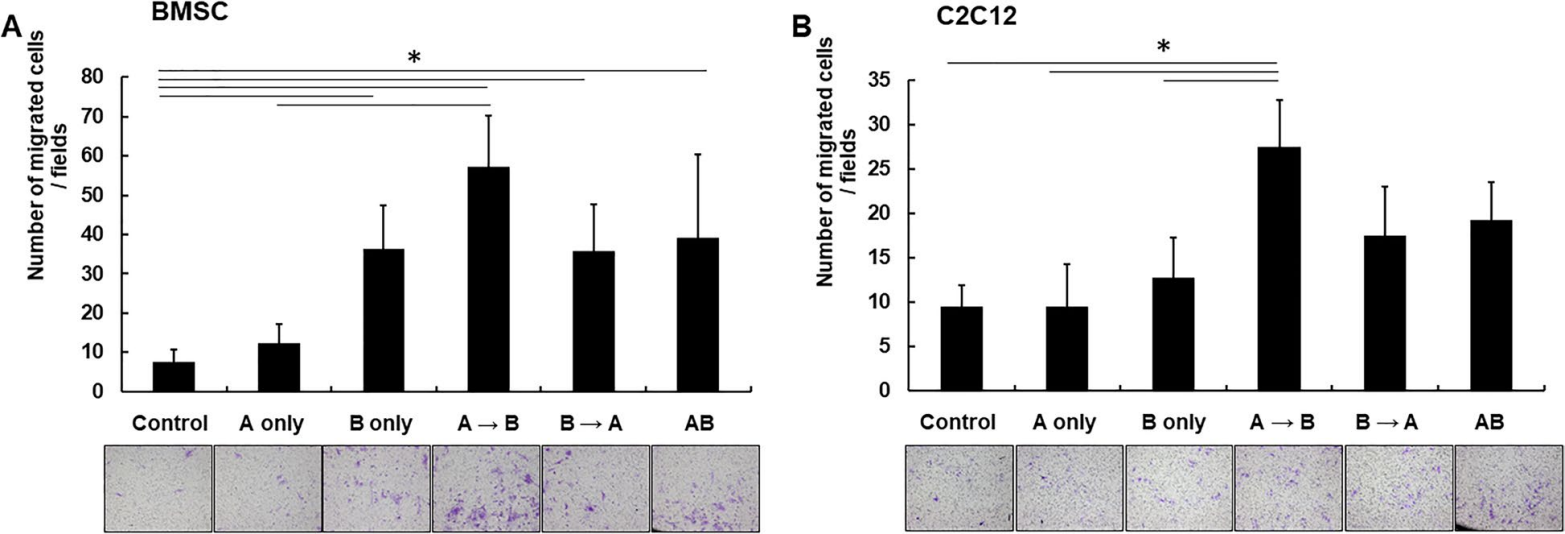
Cell migration assay to evaluate effect of combined AMD3100 and BMP-2 treatment. Migration characteristics of BMSC (**A**) and C2C12 (**B**) during treatment with AMD3100 and/or BMP-2 (control: non-treatment; A only, treated AMD3100 only; B only, treated BMP-2 only; A → B, cells pretreated with AMD3100 followed by BMP-2 treatment; B → A, cells pretreated with BMP-2 treatment followed by AMD3100 treatment; AB, denotes concomitant treatment with the two cytokines. Data indicate mean ± SD, (Upper) Quantification of number of migrated cells per fields using i-solution software in three independent experiments. (Lower) Representative microscopic image of transwell cell migration in various settings of AMD3100 and BMP-2 application (40x). Sequential AMD3100 and BMP-2 treatment (A → B group) increased mesenchymal cell migration than did AMD3100 treatment alone (both BMSC and C2C12, *p* < 0.01) or BMP-2 treatment alone (C2C12, *p* < 0.01). However, the assay results of concomitant AMD3100 and BMP-2 treatment (group AB) were not significantly different from those of BMP-2 treatment (B only) or sequential AMD3100 and BMP-2 treatment (A → B or B → A group). *Statistical difference between the groups (*p* < 0.05)

The ability of AMD3100 and/or BMP-2 to induce mesenchymal cell migration was evaluated with a migration assay. The results showed that sequential AMD3100 and BMP-2 treatment (A → B group) had a greater impact mesenchymal cell migration than did AMD3100 treatment alone (A only, in both on BMSC and C2C12, *p* < 0.01) or BMP-2 treatment alone (B only on C2C12, *p* < 0.01). However, the assay results of concomitant AMD3100 and BMP-2 treatment (group AB) were not significantly different from those of BMP-2 treatment (B only) or sequential AMD3100 and BMP-2 treatment (A → B or B → A group, *p* > 0.05) (Fig. 3).

### In vivo effect of continuous IP injection of AMD3100 on BMP-2-induced bone regeneration

**Fig. 4:**
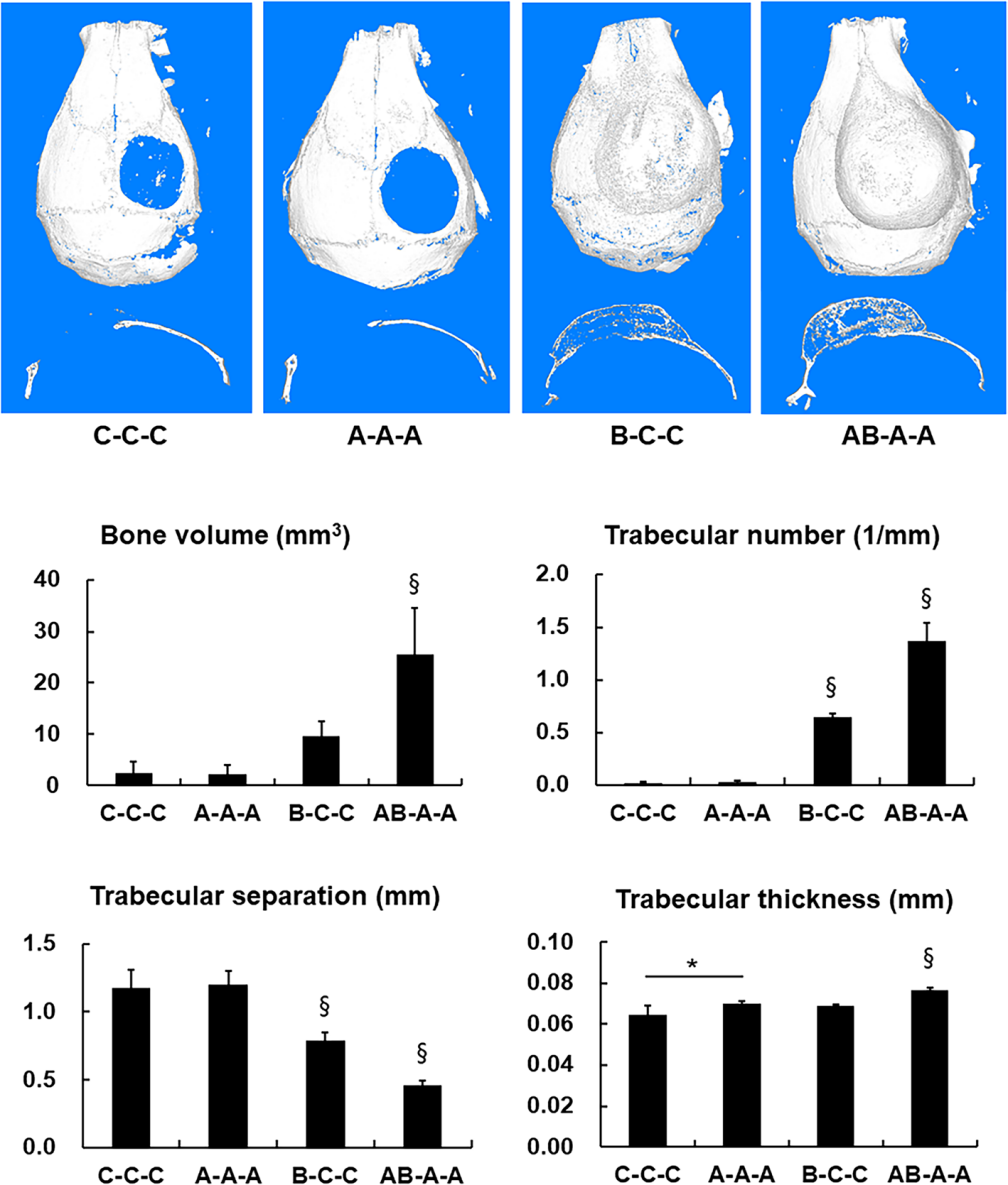
Micro-CT analysis of analysis of bone formation by continuous AMD3100 intraperitoneal (IP) injection during BMP-2-induced bone regeneration in critical-size calvarial defect. Three-dimensional micro-CT images after 28 days of PBS- o rBMP-2-loaded collagen scaffold implantation combined with or without continuous AMD3100 IP injections. Group C–C-C, PBS-loaded scaffold was inserted in the calvarial defect, and PBS was administered by IP injections on postoperative days 3 and 6; group A-A-A, PBS-loaded scaffold was implanted, and AMD3100 was administered by IP injections 1 h before the surgery with additional injections on days 3 and 6; group B-C–C, BMP-2-loaded scaffold was implanted, and PBS was administered by IP injections on days 3 and 6; group AB-A-A, BMP-2-loaded scaffold was implanted, and AMD3100 was administered by IP injections 1 h before the surgery with additional injections on postoperative days 3 and 6. Bone regeneration in a mouse calvarial defect model was evaluated with micro-CT (Upper). Quantification of new bone volume, trabecular number, trabecular separation, and trabecular thickness were shown (Lower). Mouse models with BMP-2-loaded scaffold in the defect and received continuous IP injection of AMD3100 (group AB-A-A) showed significantly higher new bone volume, trabecular number, and dense trabecular separation than those with BMP-2-loaded scaffold implantation without AMD3100 injection (group B-C–C). Data represent mean ± SD (§ significantly different from the other groups, *p* < 0.01; * significant differences among the groups, *p* < 0.01)

The effect of continuous IP injection of AMD3100 on BMP-2-induced bone formation was tested under various conditions of AMD3100 and/or BMP-2 administration. According to the micro-CT results, injection of AMD3100 three times without the BMP-2-loaded scaffold (group A-A-A) did not induce bone formation. However, continuous injection of AMD3100 three times with the BMP-2-loaded scaffold (group AB-A-A) resulted in significantly higher bone volume and trabecular number (*p* < 0.01). Trabecular separation analysis also showed that the AB-A-A group demonstrated the highest bone density when compared with all the other groups, including the experimental group with BMP-2-loaded scaffold without IP injection of AMD3100 (group B-C–C) (*p* < 0.01) (Fig. 4).

**Fig. 5:**
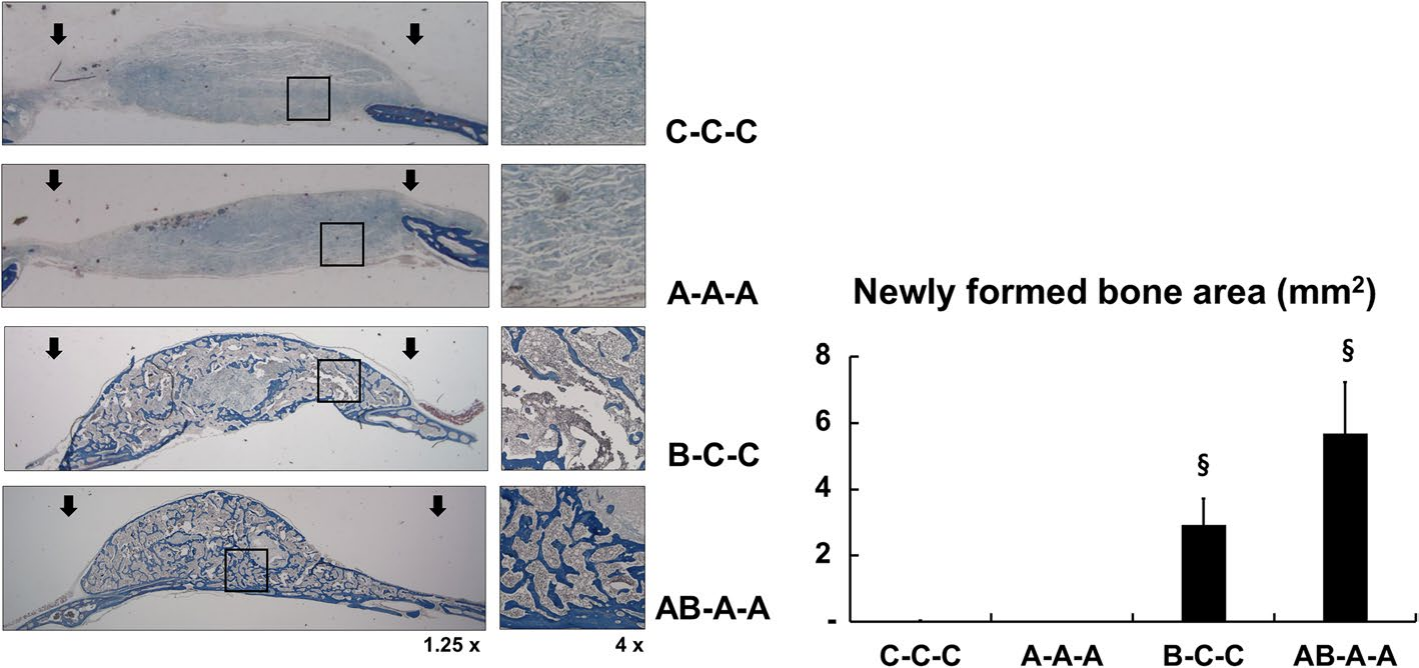
Histomorphometric analysis of bone formation after continuous IP injection of AMD3100 on BMP-2-induced bone regeneration. After micro-CT analysis, histologic analysis with trichrome histological anaysis was conducted to evaluate bone regeneration. (Left) Representative trichrome staining images of calvarial bone sections of each group. Histological sections with 1.25 × and 4 × magnification from each group are shown. (Right) Quantitative analysis of new bone formation area was performed using i-solution software. Continuous administration of AMD3100 to animals with BMP-2-loaded scaffold in the defect (group AB-A-A) show significantly higher new bone area than do any other groups (groups C–C-C, A-A-A, or B-C–C); this result is similar to that of the micro-CT analysis. Data represent mean ± SD (§ significantly different from the other groups, *p* < 0.01)

Histomorphometric analysis also showed that continuous AMD3100 treatment of the BMP-2-loaded scaffold in the calvarial defect (group AB-A-A) resulted in the most abundant new bone formation (*p* < 0.01) (Fig. 5). These micro-CT and histological analyses showed that additional continuous AMD3100 treatment reinforced BMP-2-induced bone formation.

### In vivo effect of *s*equential treatment of IP injection of AMD3100 followed by BMP-2 injection into the scaffold in calvarial defect

**Fig. 6:**
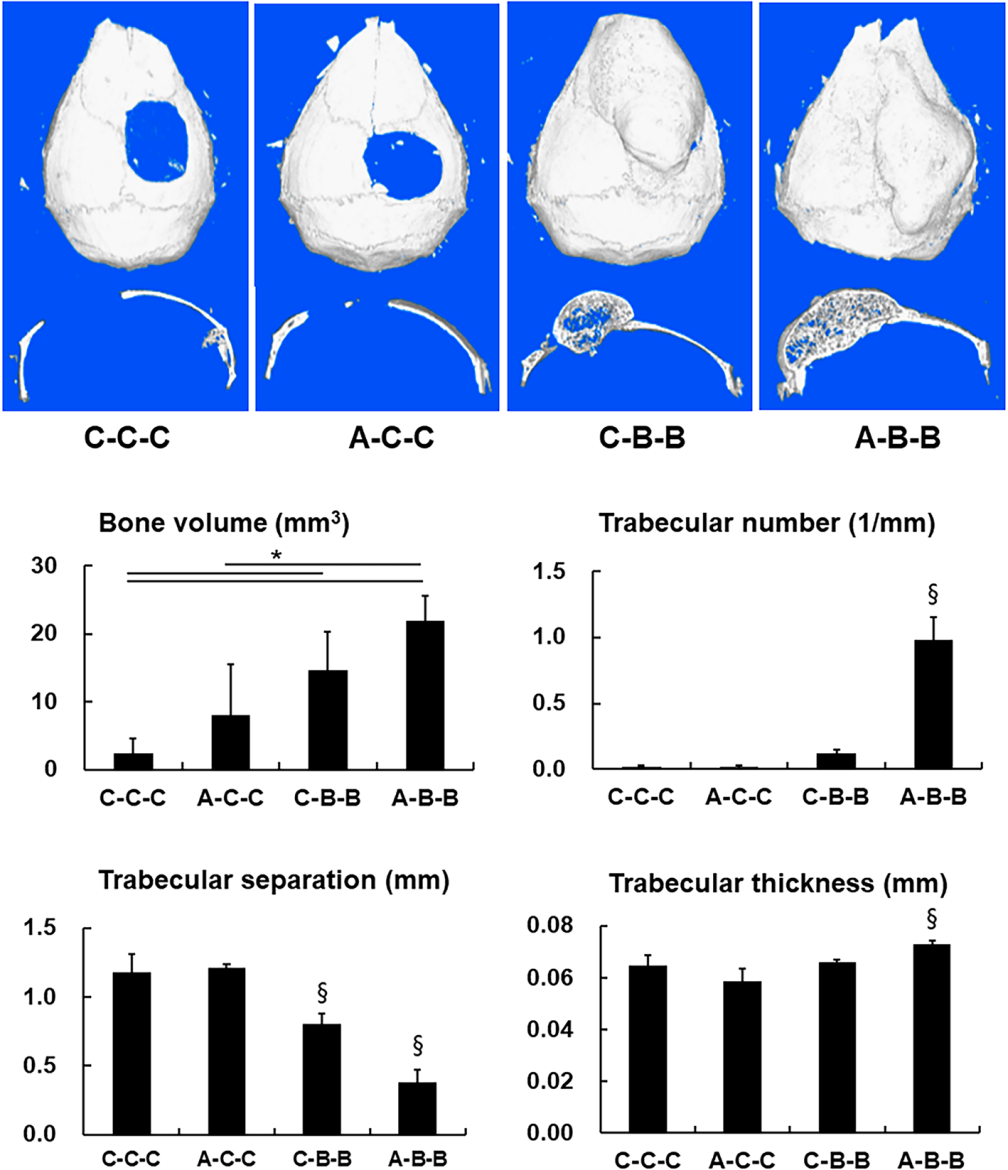
Micro-CT analysis of bone formation by *s*equential treatment with AMD3100 and BMP-2. (Upper) Three-dimensional micro-CT images of mouse calvarial defects after sequential treatment with AMD3100 and BMP-2. (Lower) Quantification of newly regenerated bone volume, trabecular number, trabecular separation and trabecular thickness. Micro-CT results show that as observed in the control group (group C–C-C), single intraperitoneal (IP) injection of AMD3100 did not enhance bone formation (group A-C–C). However, models injected with BMP-2 on the 3^rd^ and 6^th^ days after creation of the calvarial defect and implantation with the empty scaffold show significantly higher bone volume (group C-B-B). Administration of AMD3100 by IP injection on the operation day and subsequent BMP-2 injections into the scaffold in the calvarial defect on postoperative days 3 and 6 proved to be the most effective regimen (group A-B-B). Compared with continuous BMP-2 treatment alone (group C-B-B), sequential treatment of AMD3100 and BMP-2 (group A-B-B) accelerates BMP-2-induced bone regeneration. Data represent mean ± SD (§ significantly different from the other groups, *p* < 0.01); * significant differences among the groups, *p* < 0.01)

Micro-CT results showed that a single IP injection of AMD3100 did not enhance bone formation (group A-C–C). However, injection of BMP-2 on days 3 and 6 after creation of the calvarial defect and implanted with an empty scaffold showed significantly higher bone volume (group A-B-B) (*p* < 0.01). IP injection of AMD3100 on the operation day and subsequent BMP-2 injection on postoperative days 3 and 6 into the scaffold in the calvarial defect was the most effective regimen. Compared with continuous BMP-2 treatment alone (group C-B-B), sequential treatment with AMD3100 and subsequent BMP-2 (group A-B-B) accelerated BMP-2-induced bone regeneration (*p* < 0.01), which was consistent with the in vitro results. Moreover, regarding trabecular separation, pretreatment with AMD3100 resulted in an increased quantity of regenerated bone and higher density of regenerated bone (*p* < 0.01) (Fig. 6).

**Fig. 7:**
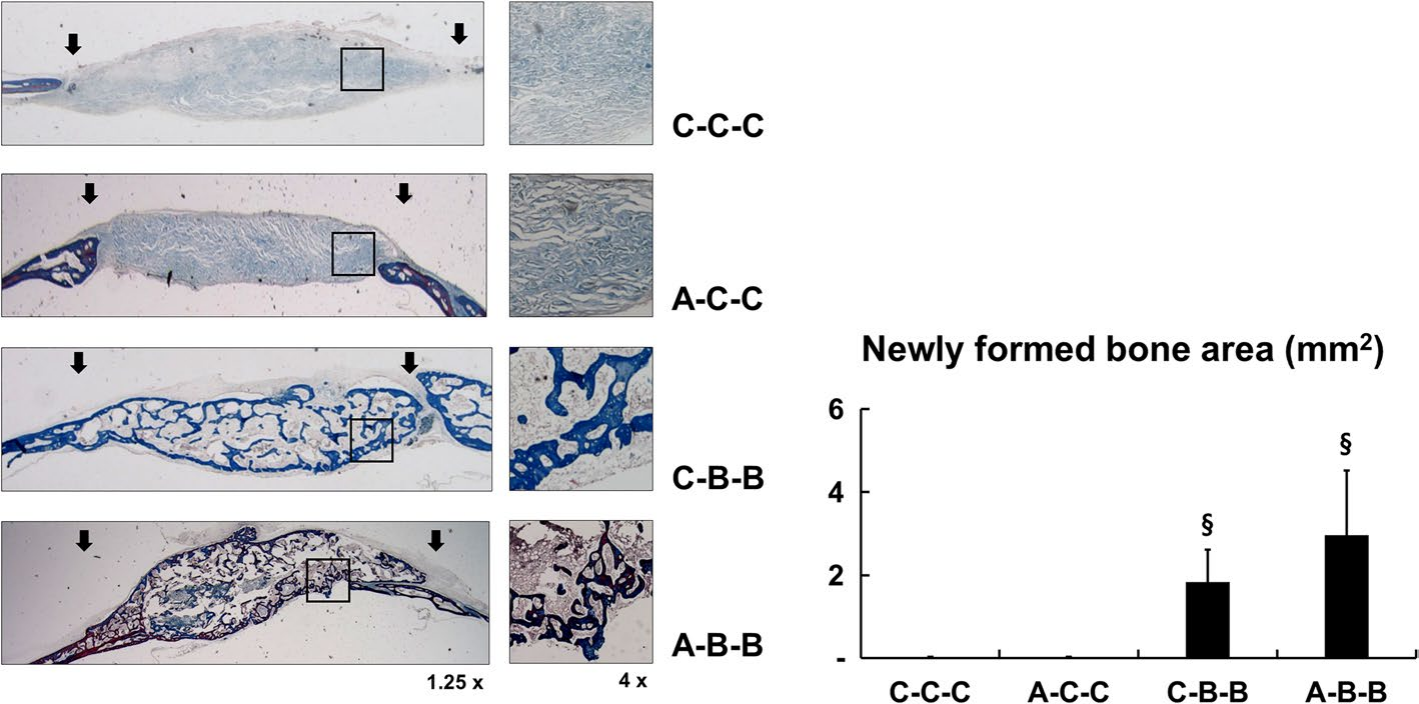
Histomorphometric analysis of bone formation after *s*equential treatment with initial AMD3100 and subsequent BMP-2. After micro-CT analysis trichrome histological staining analysis was conducted to evaluate bone regeneration. Bone formation area images were analyzed using i-solution software. (Left) Trichrome staining of representative histological Sect. (1.25 × and 4 × magnification). (Right) New bone formation areas were quantitatively analyzed using i-solution software. Models receiving single AMD3100 intraperitoneal (IP) injection at the time of calvarial defect creation surgery and subsequent injection of BMP-2 into the phosphate-buffered saline (PBS)-loaded scaffold in the defect (group A-B-B) show significantly higher number of new bone areas than do any other groups (groups C–C-C, A-C–C, or C-B-B). Data represent mean ± SD (§ significantly different from the other groups, *p* < 0.01)

Histological evaluation of the newly formed bone also showed that the sequential treatment with AMD3100 and subsequent BMP-2 resulted in significantly higher bone formation area (*p* < 0.01) (Fig. 7). These results showed that sequential treatment with AMD3100 and subsequent continuous BMP-2 induction (group A-B-B) was proved to be an effective strategy in bone regeneration, and this result was consistent with that of the micro-CT analysis.

## Discussion

The aim of the study was to evaluate whether combined treatment with a chemokine AMD3100, as a stem cell mobilizer, and BMP-2 can enhances bone regenerations in mouse calvarial defect model. We hypothesized that application of concomitant or sequential application of AMD3100 and BMP-2 would enhance more increased bone formation than control or BMP-2-only treated group. The result suggested that single or continuous injection of AMD3100 can further activate bone regeneration induced by BMP-2 application in animal model.

AMD3100 was FDA-approved for HSPC mobilization in combination with G-CSF in patients with NHL and multiple myeloma after the completion of phase III clinical trials in December 2008. Although AMD3100 does not induce direct osteogenic differentiation or calcium deposition of HSPCs in vitro, it results in greater proliferative capacity and angiogenic potential in mobilized cells than those in steady-state circulating cells [31, 32, 37].

Previous studies have shown that AMD3100 decreases ALP activity and osteocalcin production, inhibits bone nodule mineralization, and reduces the expression of Runt-related transcription factor-2 (Runx2) and osterix by inhibition of the SDF-1/CXCR4 axis during BMP-2-induced osteogenic differentiation [16, 40]. These studies primarily focused on the ability of AMD3100 to act as a CXCR-4 antagonist. Hosogane et al. [16] showed that BMP-2-induced ALP activity is decreased by blocking the SDF-1/CXCR4 signal axis with AMD3100 in mouse and human MSC cultures. In these experiments, however, cytokines were administered together (AMD3100 was administered first, followed by BMP-2 after 2 h) and the human bone marrow-derived MSCs were incubated for 7 d in the media with mixed cytokines. Conversely, in our experiments, the cells were incubated sequentially in the cytokines (every 2 days).

Zhu et al. [22] showed that AMD3100 decreases osteocalcin synthesis in BMP-2-induced osteogenesis in vitro, although it is worth noting that the experiments were performed in the presence of pertussis toxin to inhibit G-protein coupled CXCR4 receptors, which could influence cytokine production or interaction between cytokines. Wei et al. [28] demonstrated that additional AMD3100 treatment partially inhibits Runx2 expression in vitro, although the potential impact of using cells infected with myc-tagged retroviral vector in these experiments is presently undetermined. However, the results of these in vitro study could not be directly translated to in vivo condition, therefore, further animal experiment result was needed.

Some studies have revealed that single or continuous AMD3100 and SDF-1 treatment ultimately enhances BMP-2-induced bone formation in animal model [18, 31, 32]. Wise et al. [18] showed that a single IP injection of AMD3100 potentiates BMP-2 induced bone formation by stimulating the trafficking of mesenchymal cells to subcutaneous implant sites in backs of the rats 1 h before surgical implantation of absorbable collagen sponges loaded with BMP-2 on the 56^th^ day. However, continuous (weekly) AMD3100 treatment for 28 days resulted in significantly impaired bone formation even in the presence of BMP-2. Davidson et al. [31] reported that application of AMD3100 in a rat mandibular model promotes ossification and up-regulation of BMP-2 in defect sites. However, in contrast with our administration regimen, these authors applied AMD3100 by daily IP injections for 28 days. Wang et al. [32] also reported that continuous treatment mouse calvarial defects with AMD3100 induces neovascularization and enhances bone regeneration in the defect areas, both of which were orthotopically induced after daily IP injections of AMD3100 for 16 days. The authors also demonstrated that continuous AMD3100 treatment improves bone regeneration in a mouse calvarial defect model, and this treatment enhances neovascularization and increases circulating endothelial progenitor cells in vivo [32]. Although these results demonstrated that treatment of AMD3100 augments bone regeneration, its administration by daily IP injections is challenging in clinical settings. Since the experimental settings are different, it is not clear if the effect of a single injection of AMD3100 is comparable to that of continuous or intermittent injections on potentiation of BMP-2-induced bone formation. Compared to the previous results and our findings from in vitro and in vivo study, continuous treatment of AMD3100 would not be always beneficial to osteoblastic differentiation. These showed that the administration time and period would be also important for expecting synergistic effect of AMD3100 and BMP-2 treatment.

The effect of AMD3100 on BMP-2-induced osteogenic differentiation remains unclear. BMP-2 binds to its serine/threonine kinase receptor and activates intracellular receptor-regulated Smad proteins (R-Smads, Smad 1/5/8) and the mitogen-activated protein kinase (MAPK) components, extracellular signal-regulated kinase (Erk) 1/2, which subsequently regulates the osteoblast genes [41, 42]. Intracellular Smad and Erk pathways are major sub-pathways engaged in BMP-2-induced bone regeneration signal transduction [16]. Furthermore, previous studies have revealed that SDF-1 binds to G-protein-coupled transmembrane receptor CXCR-4, and regulates intracellular Smad and Erk pathway activation beyond the early phase of osteogenic differentiation [15, 16]. Recently, some studies have suggested that AMD3100 also promotes tissue regeneration by regulating the homing of MSCs to sites of tissue injury sites [29, 30]. Moreover, AMD3100 treatment increases both vascular and osteoblast density and upregulates BMP-2 expression [31, 32]. Based on the observation that AMD3100 treatment induces mobilization of HSPCs from the bone marrow into the circulating PB and up-regulates angiogenesis, we hypothesized that AMD3100 treatment would increase BMP-2-induced bone formation. However, because of the limitation of the current study, the further research is continued to elucidate the molecular mechanism.

In this study, to determine the ability of AMD3100 in promoting osteogenic differentiation, we evaluated ALP activity in an in vitro assay. The results demonstrated that the single or continuous treatment with AMD3100 alone was not sufficient for inducing osteogenesis in vitro. To determine the influence of AMD3100 in BMP-2-induced osteogenic differentiation in vitro, C3H10T1/2 cells were incubated in media containing AMD3100 and/or BMP-2 for 8 days, and their ALP activity and histological staining were analyzed (Fig. 2). The results showed that BMP-2-induced osteogenic differentiation increased with sequential application of BMP-2 after first inducing with AMD3100 (group A-B-B-B). However, sequential application of AMD3100 after initial BMP-2 treatment was relatively ineffective in inducing osteogenic differentiation (groups B-B-B-A, B-B-A-A, and B-A-A-A). These results imply that AMD3100 would be more effective in enhancing BMP-2-induced osteogenesis when used in the early stages of treatment regimens.

To ascertain the effect of AMD3100 on mesenchymal cell migration during BMP-2-induced osteogenic differentiation, we performed an in vitro cell migration assay (Fig. 3). The quantified results showed that sequential treatment with AMD3100 and BMP-2 induced significantly greater cell migration than BMP-2 alone. These results demonstrate that sequential AMD3100 and BMP-2 treatment increases the potential for cell migration in BMP-2-induced osteogenesis.

Based on these in vitro results, in vivo experiments were performed in a mouse calvarial defect healing model. We performed the experiments under the various treatment regimens with AMD3100 and/or BMP-2 to determine the influence of AMD3100 in orthotopic BMP-2-induced ontogenesis. The result showed that bone regeneration after continuous preoperative and postoperative AMD3100 induction with single BMP-2 treatment (group AB-A-A) was significantly greater than that after single BMP-2 induction (group B-C–C) The trabecular separation analysis indicated that additional AMD3100 treatment resulted in denser and more abundant regenerated bone in BMP-2-induced osteogenesis (Figs. 5 and 6). We compared single preoperative AMD3100 induction with continuous postoperative BMP-2 treatment (group A-B-B), and continuous postoperative BMP-2 treatment without AMD3100 induction group (group C-B-B), to identify the most effective timing for AMD3100 induction. Group A-B-B exhibited greater bone regeneration than did group C-B-B. The results show that greater bone regeneration was achieved with a single preoperative AMD3100 induction with continuous postoperative BMP-2 treatment (group A-B-B) than continuous postoperative BMP-2 treatment without AMD3100 induction (group C-B-B) (Fig. 7 & 8). These results suggest that sequential treatment with AMD3100 is effective in BMP-2-induced osteogenic differentiation.

In this study, the synergistic effect of AMD3100 in BMP-2-induced osteogenic differentiation was demonstrated both in an in vivo environment and in in vitro experiments. AMD3100 plays an important role in migration, recruitment and proliferation of mesenchymal osteogenic progenitor cells in early stages of differentiation and augments BMP-2-induced osteogenesis.

However, there are limitations in this study. The exact molecular mechanism and pathways related with synergistic effect of BMP-2-induced bone formation with AMD3100 was not clearly investigated. There might be a possible molecular mechanism influencing the SDF-1 and BMP-2 signal transduction pathways. It is possible that stem cell mobilization effect of AMD3100 can be greater than molecular signaling. Further research is needed to ascertain the exact mechanism or molecular cascade underlying the effects of AMD3100 and its interactions with other chemokines. Another limitation is that the end point of the animal experiments were 28 days of treatment. Therefore, more long term result might be mandatory to clarify potential long term complications or side effects, which is important for future clinical application in bone regeneration strategy.

## Conclusion

The current study showed that continuous AMD3100 IP injection to the animal with BMP-2 loaded scaffold in calvarial defect produced improved new bone formation than experimental groups with a BMP-2-loaded scaffold-only without AMD3100 IP injection. Additional experiment also showed that single IP injection of AMD3100 and subsequent BMP-2 injection to the scaffold in calvarial defect showed pronounced new bone formation than control groups. The results of the current study suggested that single or continuous injection of AMD3100 can potentiate BMP-2-induced osteoblastic differentiation and bone regeneration. This strategic combination of AMD3100 and BMP-2 may be a promising therapy for bone regeneration. The result would provide valuable regimen for accelerating bone regeneration.

## Data Availability

The datasets used and/or analysed during the current study are available from the corresponding author on reasonable request.

## Abbreviations

BMPs: Bone morphogenetic proteins
BMP-2: Bone morphogenetic protein-2
SDF-1: Stromal cell-derived factor-1
CXCR4: Cysteine-C motif chemokine receptor 4
MSC: Marrow stromal cell
HSPC: Hematopoietic stem and progenitor cell
G-CSF: Granulocyte colony-stimulating factor
NHL: non-Hodgkin's lymphoma
BMSC: bone marrow stromal cell
DMEM: Dulbecco's modified Eagle's medium
FBS: Fetal bovine serum
ALP: Alkaline phosphatase
PBS: Phosphate Buffered Saline
CFU: Colony-forming unit
IP: Intraperitoneal
MAPK: Mitogen-activated protein kinase
Micro-CT: Micro-computed tomography
EDTA: ethylenediamine tetraacetic acid
H&E: hematoxylin and eosin
SD: Standard deviation
Runx2: Runt-related transcription factor-2
